# Artificial intelligence-guided strategies for next-generation biological sequence design

**DOI:** 10.1093/nsr/nwae343

**Published:** 2024-09-26

**Authors:** Pengcheng Zhang, Lei Wei, Jiaqi Li, Xiaowo Wang

**Affiliations:** Ministry of Education Key Laboratory of Bioinformatics, Center for Synthetic and Systems Biology, Bioinformatics Division at the Beijing National Research Center for Information Science and Technology, Department of Automation, Tsinghua University, China; Ministry of Education Key Laboratory of Bioinformatics, Center for Synthetic and Systems Biology, Bioinformatics Division at the Beijing National Research Center for Information Science and Technology, Department of Automation, Tsinghua University, China; Ministry of Education Key Laboratory of Bioinformatics, Center for Synthetic and Systems Biology, Bioinformatics Division at the Beijing National Research Center for Information Science and Technology, Department of Automation, Tsinghua University, China; Ministry of Education Key Laboratory of Bioinformatics, Center for Synthetic and Systems Biology, Bioinformatics Division at the Beijing National Research Center for Information Science and Technology, Department of Automation, Tsinghua University, China

Recent advancements in artificial intelligence (AI) have revolutionized our ability to model biological sequences, paving the way for a new AI-driven paradigm in next-generation biological sequence design. In this article, we introduce how AI is utilized for conducting digital experiments, navigating the vast sequence landscape and elucidating the intricate connections between sequence and function through advanced generative and predictive modeling techniques. Additionally, we discuss the adoption of active learning approaches to bridge the gap between digital simulations and wet-lab experiments, thereby significantly improving efficiency in testing the most informative data to elevate the performance of AI models.

Synthetic biology aims to obtain desired functions by reprogramming the genetic code of living systems [1]. Central to this discipline is the design of biological sequences—DNA, RNA and proteins that govern various life activities. The high complexity and high-dimensional regulatory characteristics of these sequences pose significant challenges to their design. For instance, a DNA or RNA sequence of 1000 base pairs could theoretically have over 10^600^ possible arrangements. However, functional sequences that adhere to biological constraints may only occupy a small subset of this immense sequence space. Moreover, the relationship between sequence and function is notably non-linear [2], complicating the mapping of these relationships with limited data sets. It is impractical to explore the whole sequence space by costly and time-consuming trial-and-error experiments. Traditional design methods, which either rely on human understanding of biological codes [1] or involve evolutionary strategies that start from naturally functional sequences [3], are confined to exploring only a limited portion of the possible sequence space. This limitation restricts our ability to discover and *de novo* design biological sequences with desired functions, particularly those that are not observed in nature. Rational design approaches for biological sequences are urgently needed.

Recently, AI, especially deep learning, has been proven to be more effective at uncovering patterns, relationships and mechanisms from vast amounts of data [4]. This capability has opened up new opportunities to harness the power of AI for next-generation biological sequence design. AI approaches have been developed to perform digital experiments, typically consisting of a generative model that creates candidate sequences and a predictive model that evaluates the properties of these sequences. These models iteratively optimize sequences in the digital space and can be integrated with biological experiments for validation and/or model refinement (Fig. 1A). This article will highlight the concepts, advancements and ongoing challenges in AI-guided biological sequence design.

**Figure 1.**
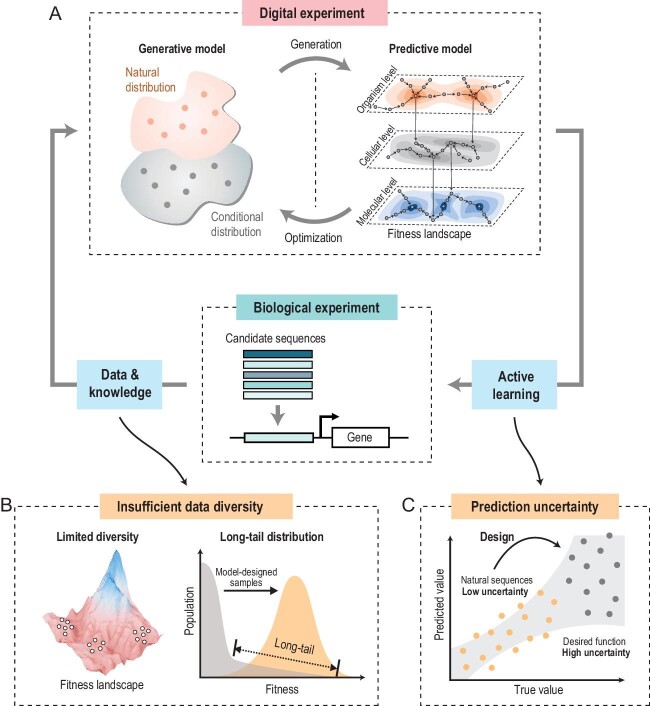
(A) Schematic representation of AI-guided biological sequence design approaches. These approaches typically utilize generative and predictive models to perform digital experiments for accelerating the evolutionary process in the digital space. The integration of digital experiments and biological experiments through active learning improves the efficiency and accuracy of biological sequence design. (B) Insufficient data diversity and the long-tail distribution in training datasets limit model performance. Conducting additional biological experiments, guided by AI models, can help address these long-tail distribution challenges. (C) The role of prediction uncertainty is crucial in active learning strategies for selecting information-rich sequences to test.

## BIOLOGICAL SEQUENCE DESIGN WITH DIGITAL EXPERIMENTS

Natural life systems acquire biological sequences with desired functions through a lengthy evolutionary process. This process, which can span thousands or even millions of years, involves two key steps: the generation of variants and the selection of these variants based on their fitness. In contrast, AI-based methods are developed to perform digital experiments to accelerate this process through fast and repetitive iterations within the digital space. AI-guided digital experiments typically utilize generative and predictive models to achieve this goal. Generative models use natural or functional synthetic samples to estimate the distribution of functional sequences in the whole sequence space, and sample from this distribution to obtain candidate sequences [5]. Predictive models try to map the sequence–function relationships, serving as a fitness landscape [2]. This landscape can be multi-level, allowing for the extraction of patterns in which biological sequences function at the molecular, cellular and organismal scales [6]. The predictive models evaluate the candidate sequences generated by the generative models, and the candidates predicted with good properties can then be iteratively used as prompts to guide the generative models [7]. Such digital experiments transfer the variant generation and fitness selection processes of natural evolution into the digital space, enabling the rapid and efficient exploration of the huge sequence space. For a comprehensive overview of the diverse applications of AI models in biological sequence design, readers can refer to recent reviews [8–10].

This iterative approach between generative and predictive models is crucial for the effectiveness of digital experiments. Relying solely on predictive models can lead to issues such as unreliable predictions, local minima and difficulty in achieving high diversity in the sequences [11]. Generative models can produce candidate sequences that conform to the constraints of functional sequences, and using these as inputs for predictive models helps improve prediction accuracy and enables the learning of sequence–function landscapes from smaller subspaces. Conversely, relying solely on generative models alone may not yield sequences that enhance functionality. At this point, the sequence–function landscape learned from predictive models becomes crucial for guiding improvements. Currently, various machine learning methods have been utilized to facilitate this iterative approach, such as the genetic algorithm [12], reinforcement learning [13] and Bayesian optimization [14].

Recent studies have demonstrated the potential of digital experiments in designing biological sequences. For instance, Ingraham *et al.* proposed the Chroma framework for high-quality protein generation, with the crystal structures of two designed proteins showing atomic-level consistency [15]. Zrimec *et al.* developed a deep learning model for designing regulatory DNA sequences, finding that 57% of them had higher expression levels than natural sequences [16]. Sumi *et al.* used a variational autoencoder (VAE) and a covariance model (CM) to generate the *glmS* ribozyme, achieving a high design success rate and enhancing the activity compared to natural sequences [17].

Although AI models excel at learning common sequence patterns from large data sets, they may struggle to capture rare but crucial patterns that appear only in a small subset of data points, or even in a single known sequence. In such cases, integrating biological prior knowledge into generative models to learn the conditional probability distribution of sequences is an effective strategy. For example, we employed a conditional generative adversarial network (cGAN), incorporating transcription factor binding sites (TFBSs), to design doxycycline-inducible promoters, resulting in 72.2% of designed promoters showing improvements in both induced activity and activation rate [12]. Watson *et al.* presented the RoseTTAFold diffusion (RFdiffusion) model, which uses functional motifs and enzyme active site scaffolding to conditionally design proteins, demonstrating impressive performance [18]. Chu *et al.* proposed a language model that incorporated the secondary structure and minimum free energy to optimize ribosome loading of the 5′ untranslated region of RNA sequences [19]. These paradigms of integrating data with knowledge expand the performance and application scope of digital experiments.

## INTEGRATING DIGITAL AND BIOLOGICAL EXPERIMENTS FOR MORE EFFICIENT DESIGN

The performance of AI models in designing biological sequences is constrained by the limited diversity of training data (Fig. 1B). Natural biological sequences, the primary source for these models, are insufficient compared to the expansive sequence space, leading to a lack of thorough exploration [11,20]. Moreover, the predominance of biological sequences with zero or extremely low functionality leads to a long-tail distribution (Fig. 1B), resulting in an extreme imbalance in the training data sets and causing AI models to often recognize inaccurate sequence patterns. Wittmann *et al.* demonstrated that reducing the inclusion of low-fitness protein variants in training data sets can help improve the optimized fitness of proteins [20].

Supplementing training data sets with sequences that extend beyond naturally occurring genomic sequences enables the exploration of a larger sequence space [21]. The integration of digital experiments and biological experiments provides an effective solution to the challenges of obtaining diverse sequences for model training. By experimentally testing candidate sequences and subsequently retraining, the performance of AI models can be significantly improved. This iterative approach ensures that the models are continuously refined and progressively approximate real-world observations. For instance, Wang *et al.* measured the activities of promoter sequences designed by their model in cells and used this data to retrain their predictive model. This approach significantly increased the success rate of promoter design from 45.8% to 70.8% [22]. Friedman *et al.* actively selected generated sequences for testing and trained the model using results from multiple rounds of massively parallel reporter assays (MPRAs), demonstrating that informative sequences help to improve model accuracy [23].

How to select sequences for experimental testing is a key issue in the integration of digital experiments and biological experiments. Developing an effective active learning strategy is a powerful approach for iteratively exploring sequence space. This method has been widely applied in virtual drug screening and in capturing compound–ligand interactions, significantly improving the efficiency of sequence space exploration [23,24]. One effective approach is machine learning-assisted directed evolution, a form of active learning that uses predictive models to identify and prioritize promising variants from vast libraries of potential sequences, allowing researchers to focus on candidates most likely to achieve desired functions before conducting resource-intensive experiments [7]. Another form of active learning involves selecting sequences based on the model's uncertainty to maximize information gain (Fig. 1C). This method focuses on sequences for which the predictive model shows the highest uncertainty, as testing these sequences provides the most valuable data for improving model performance. Hie *et al.* adopted the Gaussian process to quantify prediction uncertainty and utilized this uncertainty to guide the design of biological experiments, successfully enhancing the generalization ability of AI models and accelerating protein design [24]. When high-throughput screening is time-consuming and costly, the active learning strategy is particularly valuable, as it can integrate digital and biological experiments to effectively explore the sequence space and enhance model performance.

## CONCLUSIONS AND DISCUSSION

AI-guided strategies are transforming the paradigm of engineering biological sequences. The integration of digital experiments with biological experiments effectively addresses the challenges posed by the limited diversity of training data, greatly improving the precision and reliability of AI models. This approach has significantly advanced the efficient exploration of sequence space and the *de novo* design of biological sequences. Biological sequences interact dynamically with complex, multi-level biological environments, exhibiting a wide range of functions [1]. To better capture this complexity, future research should focus on developing high-throughput experimental methods that provide more comprehensive information, along with multi-scale, spatiotemporally dynamic predictive models, which will enable us to understand and design biological sequences with complex functionalities and high robustness. Moreover, since AI models often operate as ‘black boxes’, developing interpretation methods to extract explicit biological rules and building explainable AI models based on these insights is an important future direction. Understanding the underlying principles of AI models is also crucial for their application in scenarios requiring high safety, such as gene therapy and agriculture.
